# Roadmap Out of COVID-19

**DOI:** 10.5704/MOJ.2011.002

**Published:** 2020-11

**Authors:** J Thor, E Pagkaliwagan, A Yeo, J Loh, C Kon

**Affiliations:** Department of Orthopaedic Surgery, Changi General Hospital, Singapore

**Keywords:** COVID-19, trauma, orthopaedics, Singapore, pandemic

## Abstract

The recent coronavirus disease (COVID-19) was declared as a public health emergency by the World Health Organisation on 30th January 2020, and has now affected more than 100 countries. Healthcare institutions and governments worldwide have raced to contain the disease, albeit to varying degrees of success. Containment strategies adopted range from complete lockdowns to remaining open with public advisories regarding social distancing. However, general principles adopted by most countries remain the same, mainly to avoid gatherings in large numbers and limit social interactions to curb the spread of disease. In Singapore, this disease had a very different progression. The first wave of the disease started with the confirmation of the first COVID-19 positive patient in Singapore on 23rd January 2020. Initially, the daily number of confirmed cases were low and manageable. With a rise in unlinked cases, the Disease Outbreak Response System Condition (DORSCON) status was raised from yellow to orange. New cluster outbreaks in foreign worker dormitories led to the rampant spread of disease, with daily spikes of COVID-19 cases. As of 7th June 2020, we have a total of 37,910 confirmed cases of COVID-19 infections, the highest in Southeast Asia, 12,999 active cases and a manageable mortality count of 25 deaths. This details our unique method for dealing with a pandemic, including a brief demographic of trauma patients during this period. We were able to conserve sufficient resources to ensure that our essential services can still continue. Moving on, we have to ensure the continued protection of our population, especially the vulnerable groups such as the elderly and the immunocompromised, as we reopen.

## Introduction

The novel coronavirus (SARS-CoV-2) was first reported in Wuhan, China. Initially in late December 2019, cases of severe pneumonia of unknown aetiology in Wuhan, China were reported to the World Health Organisation (WHO)^1^. Later known as the coronavirus disease (COVID-19), it began to spread aggressively within the Hubei region of China, proceeding to infect other regions of China and the rest of the world. This evolved rapidly into the pandemic we know today. It has forced healthcare systems worldwide into a state of hyper efficiency, with no choice but to optimise all the limited medical resources available. This has posed a challenging problem for the Singapore healthcare system as well.

The first case of confirmed COVID-19 infection arrived on our shores on 23rd January 20202, a Chinese national from Wuhan. A swift response was mounted by the local authorities, but alas the number of cases increased, especially unlinked clusters. Hence, Disease Outbreak Response System Condition (DORSCON) level^3^ was raised from yellow to orange on 7th February 2020^4^, signalling community spread but that it was still contained.

While other countries such as Italy, Spain and France implemented complete lockdowns^5^, Singapore chose to adopt a different approach, terming it the 'circuit breaker'. Starting from 7th April, all schools and non-essential services were closed, until 1st June 2020, in an attempt to curb the spread of disease in the community. However, individuals were still allowed to leave the house under various extenuating circumstances, such as to care for their elderly relatives and to exercise in open areas. In early April, the number of confirmed cases rose rapidly to several hundred daily due to the emerging clusters in the foreign worker dormitories^2^. The spread of disease amongst foreign workers progressively worsened, leading to a large and rapid increase in COVID-19 infections locally. However, during this period, the number of COVID-19 infections in the community remained low. As of 7th June 2020, the number of active COVID-19 cases in Singapore was 12,999 and there were 25 deaths from COVID-19 complications^6^. The government also sourced for creative and alternative housing options for locals and foreign workers alike, who were facing quarantine or serving stay home notices. Cruise ships, hotels, sports halls, vacant public housing and even the Singapore Expo were turned into novel temporary housing alternatives^7^.

**Fig. 1:**
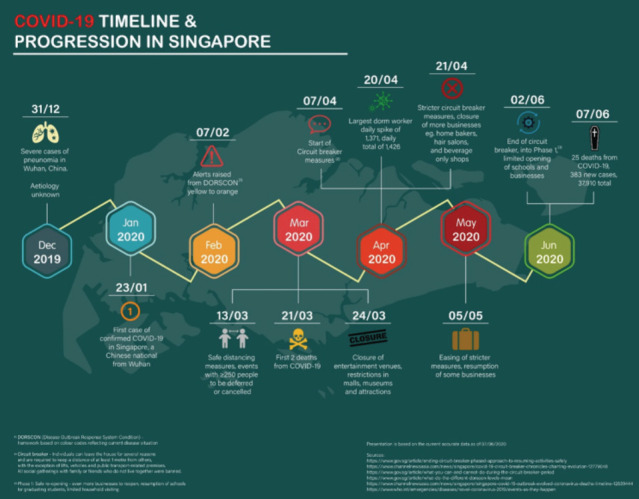
COVID-19 timeline and progress in Singapore.

A simple infographic depicting the timeline and progression of COVID-19 in Singapore has also been included (Fig. 1). A brief retrospective study of the demographics of trauma patients during this time period was done as well, to examine the difference trauma patters and trends in times of decreased mobility.

## Nationwide Healthcare System

The COVID-19 pandemic necessitated changes in our everyday healthcare practice. Hospital beds in the general ward and intensive care unit (ICU) needed to be made available, as the COVID-19 numbers increased. Most public health hospitals in Singapore are tertiary hospitals capable of handling trauma, however the most complex trauma cases were routed to the larger hospitals, with no change in existing protocols.

Specialists who held multiple positions in different hospitals across the nation were barred from cross cluster travel to minimise the risk of contamination, effectively restricting them to a single primary health institution. Healthcare personnel were also subject to cancellation of their annual leave and further requests were temporarily put on hold. Potential plans for any overseas travel were denied as well, as manpower issues will arise in the event that our healthcare workers contract COVID-19.

## Hospital Reorganisation

**Fig. 2:**
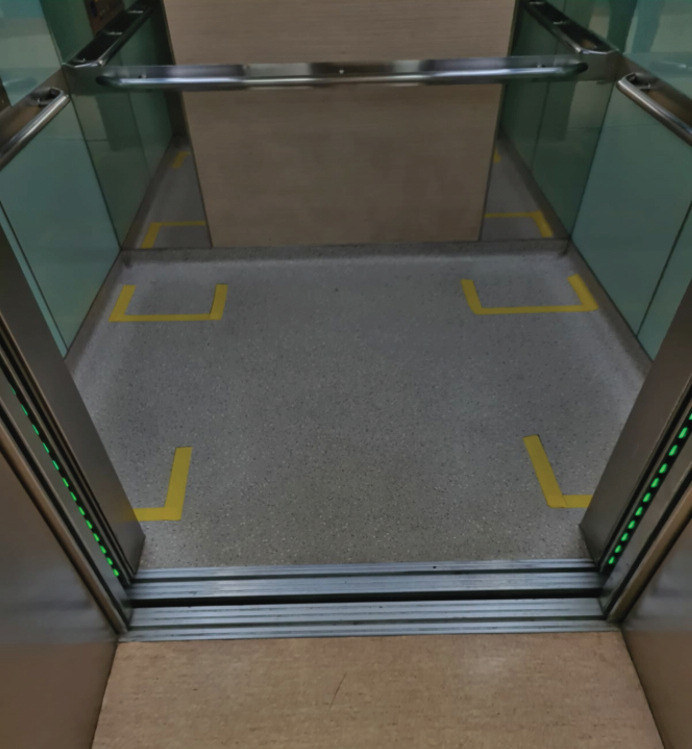
Further safe distancing measures in the lift itself, limiting maximum lift occupancy to maintain social distancing.

Various strategies were employed in our hospital to ensure the smooth workflow of medical operations, minimising risk of COVID-19 exposure to staff and patients alike, and to maintain adequate resources in a COVID-19 emergency. Multiple points of entry into our hospital were closed off, instead maintaining only three routes of entry and exit. These entries and exits were manned at all times, with staff measuring the temperature of all visitors and patients. Visitors and patients had to fill in mandatory forms for screening and contact tracing purposes. A new sticker, valid only for a day and also changed daily, was given to visitors and patients entering the hospital after they have been screened. Everyone entering and leaving the hospital was required to ‘check in’ and ‘check out’ online, including healthcare personnel working in the hospital. A government developed mobile phone application was also made mandatory for all members of the staff, allowing for community tracing via their handheld devices. Safe distancing measures were also applied to all available aspects of the hospital, such as the public areas (Fig. 2). Visitation policies were also amended, to disallow visitors from entering the general ward. Only patients deemed dangerously ill were allowed to have visitors in the wards.

All healthcare staff were required to wear surgical masks in the hospital. Different clinical areas in the hospital were classified as high-risk areas, such as the emergency department, intensive care units and high dependency wards. In these areas, protective personal equipment (PPE) and N95 masks were required at all times. Surgeons were also trained in the donning of the powered air purifying respirators (PAPRs) and PPE. All relevant healthcare personnel involved in the care of patients also underwent training for the donning and doffing of PPE, as well as the indications for PPE use. Healthcare personnel were also required to take their temperatures twice daily and then report them online. If there were any healthcare worker suspected to have had COVID-19 exposure, they were notified and immediately relieved of their duties for 14 days, allowing them to return home for self-quarantine. They were also closely monitored by the Singaporean Ministry of Health daily while on home quarantine.

Elective orthopaedic surgeries were abruptly cancelled starting from 7th February 2020, culminating in a grand total of 234 cancellations. However, in the initial stages, day surgeries were allowed to continue, a practice adopted in other healthcare institutions in Singapore as well^8^. This was because these patients would be discharged within 23 hours of their hospital admission, not requiring a significant increase in hospital resources or beds. There were also limited day surgery beds available in our institution, limiting the number day surgeries possible. By allowing elective surgery to proceed, it serves to ease the subsequent load of elective surgeries pending^9^. Also, no further elective listings were allowed, except in cases of trauma requiring surgical fixation or semi-urgent cases involving tumours needing excision or resection. As the number of active COVID-19 cases in Singapore grew, even day surgery was disallowed thereafter.

Our orthopaedic outpatient clinics were also targeted to reduce the hospital footfall in general. Patients who were previously given a six months or long follow-up appointment, and are due for review again in the coming months of March, April and May, were compiled into a list. This list then underwent multiple levels of review, firstly by the junior doctors in the department and then senior clinical staff. If patients were deemed to not require early or urgent review, their appointments were postponed for a few months. This helped to reduce the number of patients to a level that the department could cope with, even making allowances for unforeseen manpower shortages. Outpatient appointments were postponed if patients were found to have ARI symptoms. However, if their outpatient appointments were deemed urgent for clinical review, they were reviewed in a separate clinic area by staff wearing PPE.

In the operating theatre complex, a separate section of theatres was set aside for confirmed or suspected COVID-19 patients requiring surgery. Floorplans with designated pathways were created to transport patients, staff and equipment to the necessary operating theatres. A separate operating theatre was also designated as a space for surgeons, nurses and anaesthetists alike to don the PAPR gear. Announcements would also be made throughout the operating theatre complex that suspected or confirmed COVID-19 patients were arriving, to alert all theatre staff reminding them to stay at least 2m away or to avoid the area completely. During intubation and extubation of the patient, only the anaesthetist and the relevant nurses were allowed to remain in the operating theatre.

## Additional Novel Measures

The department also went the extra mile to further segregate the different teams to prevent further spread of COVID-19 in the event that a member of the team gets infected. This way, it reduces the possibility of cross contamination. Members of the staff were reassigned to different working areas of the hospital to replace the usual common orthopaedic surgery clinical staff office. Previously, our orthopaedic department was divided grossly into 4 large teams with more than 10 members in every team. Due to COVID-19, these large teams were further subdivided to proportionate numbers of junior and senior staff to form multiple smaller, functional units. For example, the spine surgery team was subdivided into two smaller teams, allowing for the continued provision of essential services at all times.

**Fig. 3:**
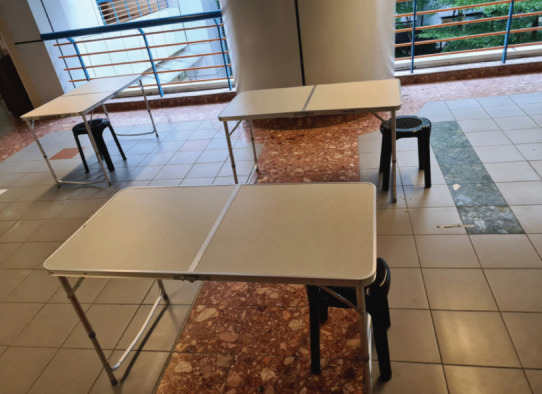
Further segregation measures during mealtimes in public areas to maintain social distancing.

Further segregation measures were established gradually. Everyone was required to avoid congregation in numbers, especially during mealtimes. People were encouraged to have their mealtimes alone, or at least sit 1m apart from each other (Fig. 3). Previously held internal departmental audit meetings and teaching sessions were all moved to online platforms, enabling the medical staff to attend without physically congregating in large numbers, reducing the risk of transmission to the department.

Upon arrival in the emergency department, all patients underwent screening. Patients with fever and/or acute respiratory infection (ARI) symptoms were sent to an isolation ward. From there, they were swabbed to determine COVID-19 status, and they were de-isolated if two swabs taken on different days were negative. If they were found to be COVID-19 positive, they were sent to another ward with the other COVID-19 positive patients for segregation. Postoperative patients with spiking fevers were also subsequently isolated in a separate ward, classified as low risk, until two swabs returned, confirming that they were COVID-19 negative. A special 24 hours COVID-19 hotline was also set up in the hospital, allowing for consultation with an infectious diseases specialist if any doubts should arise with regards to the need for swabbing or isolation.

**Fig. 4:**
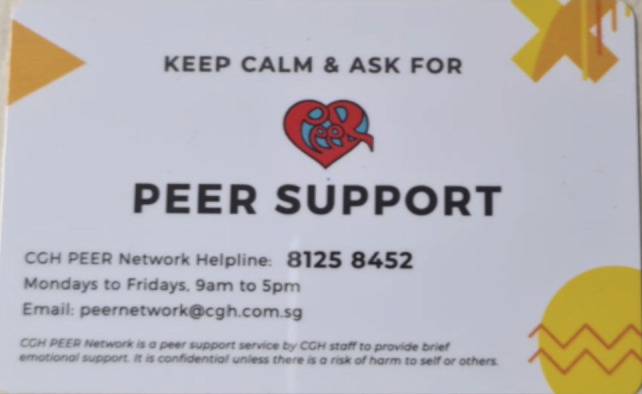
Various methods for healthcare staff to seek psychological help.

The psychological impact of the COVID-19 crisis should not be taken lightly as well. Seeing as our healthcare personnel are on the frontlines, it is undeniable that doubts and worries of contracting or even spreading the disease to their loved ones will weigh heavily on their mind. Studies in China have demonstrated that the medical staff working in the epicentre of the COVID-19 pandemic scored significantly higher on all indicators of psychological stress compared to college students^10^. A dedicated hotline was set up for healthcare workers facing significant stressors in the care of COVID-19 patients or other related issues, allowing them proper access to psychological help in these trying times (Fig. 4).

## Different Patient Groups

### Prisoners

Our institution is the only one in Singapore facing this unique group of patients. Prisoners are an isolated cohort of patients, away from the COVID-19 pandemic. However, it is important that patients who return to prison after their hospitalisation are not COVID-19 positive, or it will lead to catastrophic spread within the prison compound. All the patients arriving from prison are allocated to a bed in the prison ward. If they are symptomatic, they are then isolated until proven COVID-19 positive or negative. A criteria for discharge back to prison includes a swab taken within 72 hours of planned discharge. Prisoners requiring outpatient clinic review were given a separate clinic session on Saturdays, with no other patients in the same session, to prevent cross contamination.

### Foreign workers

The COVID-19 infection rates amongst foreign worker residents living in dormitories spiked in early April, peaking in late April. High daily rates of confirmed COVID-19 cases were mostly detected in the foreign worker dormitories, while local and imported cases have significantly dropped during the circuit breaker period. Foreign workers with fever or ARI symptoms were isolated and swabbed to test for COVID-19. Swabs were also done within 72 hours of planned discharge to ensure that the patient was confirmed COVID-19 negative. These measures were set in place to prevent more foreign worker clusters. Similar to the Saturday clinics for prisoners requiring outpatient review, separate clinic sessions were set up for the foreign worker population as well.

## Our Local Experience

From January to April 2020, there have been 402 trauma cases having undergone surgery in Changi General Hospital. These patients include those with multiple and complex injuries requiring multiple operations. While it appears that most hospitals worldwide have adopted similar policies with regard to change in operating procedure as well as operating theatre protocols^11^, we have targeted and implemented stricter protocols due to our unique situation in Singapore along with the different patient cohorts in our institution.

Despite the implementation of the circuit breaker and a change in overall mobility trends^12^, implying that more people stay at home and spend less time in public places during this period, the trauma load seen in our hospital remains significant. During this period, lower limb trauma predominates, at 68.9%. On the contrary, New Zealand have reported different results, mainly showing a decrease in trauma load and severity of trauma^13^.

In our tertiary hospital with a relatively high trauma load, we have managed to keep our staff symptom free and nobody has tested positive for COVID-19. This enabled us to maintain our manpower to cope with the trauma load. In contrast, up to 20% of the healthcare workers contracted COVID-19 in their line of work in Lombardy, Italy^14^. The National Health Commission of the People’s Republic of China have also reported a total of 3387 healthcare workers infected with COVID-19, as of 3rd April^15^. Patients requiring urgent or semi-urgent surgery were still able to receive proper treatment required. Early access to surgery also helped to reduce the burden on the existing hospital resources and obtain the best possible outcomes for our patients. However, our measures may not be applicable to other countries battling COVID-19 due to our unique problem with rampant COVID-19 infections mostly isolated within the foreign worker dormitory clusters. Our mortality rates may not be comparable to other countries as well in view of the younger age group of COVID-19 patents.

## Discussion

Currently at the time of writing, Singapore stands as the country with the highest number of COVID-19 infections in the entire Southeast Asia^16^. Despite the limited healthcare resources available, we have managed to optimise resources to achieve good outcomes for our patients and to protect our healthcare staff. This achieves the aims of sustainability in the times of crisis, allowing for essential services to continue. Data available from EuroMOMO has shown a marked increase in excess deaths results from the COVID-19 pandemic, although numbers have started to come down now as the disease numbers improve^17^. Countries like Spain and Italy have seen a large increase in excess mortality from the COVID-19 problem. All of us, as physicians and surgeons alike, are in this fight against COVID-19 together as we place our patient’s well-being and health above our very own. Moving forward, as countries worldwide are attempting to reopen their economies, we risk a resurgence in COVID-19 cases as new clusters are also surfacing^18^.

## Conclusion

This is our unique Singaporean perspective and methods for handling this crisis with our limited resources. Despite having weathered the first wave and successfully flattened the curve during the circuit breaker period, this last phase will likely be the most challenging as we start to resume elective surgery and daily operations. The protection of vulnerable groups such as the elderly and immunocompromised, will have to be taken into consideration as further measures are gradually eased.
